# Development and validation of a novel index to assess the perceived impact of sports-related oro-dental trauma among adolescents: findings from Sri Lanka

**DOI:** 10.1186/s12903-023-03097-6

**Published:** 2023-06-14

**Authors:** Iresha Udayamalee, Hemantha Amarasinghe, Ping Zhang, Newell Johnson

**Affiliations:** 1 Health Promotion Bureau, Ministry of Health, Colombo, Sri Lanka; 2 School of Medicine and Dentistry, Griffith University, Gold Coast Campus, Queensland, Australia; 3 Faculty of Dental Sciences, University of Jayawardhanapura, Colombo, Sri Lanka; 4 Menzies Health Institute, Griffith University, Gold Coast Campus, QLD Australia; 5 Faculty of Dentistry, Oral and Craniofacial Sciences, King’s College, London, UK

**Keywords:** Oral health-related quality of life, Dental trauma, Sports dentistry, Impact of trauma, Unmet dental treatment need, Adolescent Oro Dental Trauma Impact Index (AODTII)

## Abstract

**Background:**

Sports-related oro- dental trauma, such as tooth fracture, displacement, mobility, and avulsion, cause significant concern among adolescent players due to detrimental impacts. The current study aims to develop, validate and assess the reliability of a simple index as a questionnaire to assess the impact of sports-related oro-dental trauma both untreated and treated, among adolescent school children in Sri Lanka.

**Methods:**

AODTII, an adolescent oro-dental trauma impact index, was developed and validated using a mixed-method approach. Items for the index were generated by quantitative as well as qualitative analysis of the results from Oral Health-Related Quality of Life Questionnaires, personnel interviews with experts and focus group discussions with adolescents. Principal component analysis and Exploratory factor analysis were used to create the index. The index was validated in the Sinhala language, and the reliability of the index was assessed using a separate sample in the school context in the Colombo district.

**Results:**

The initial list of 28 items was reduced to 12 by the Principal Component Analysis. Exploratory Factor Analysis categorised the variables into four latent constructs; physical impact, psychosocial effect influenced by peer pressure, the impact of oral health care and the impact caused due to unmet dental trauma treatment need. The cut-off values of the AODTII were based on PCA. The index achieved the Content Validity Ratio of 88.33. The construct validity was assessed with confirmatory factor analysis by developing a structural equation model. It obtained good model fit indices of RMSEA value of 0.067, SRMR of 0.076, CFI of 0.911 and the Goodness of Fit index of 0.95. The homogeneity was ensured with convergent and discriminant validity. The Cronbach's alpha value was 0.768, ensuring reliability. The index assesses the level of impact due to oro- dental trauma and identifies whether the adolescents perceive it significantly or not.

**Conclusion:**

Twelve-item AODTII emerged as a reliable and valid tool to assess the perceived impact of untreated and treated sports-related oro- dental trauma on Sri Lankan adolescents with implications for its use in other populations. Further research is required to improve the translational value of AODTII. Moreover, the tool is potential as a patient-centred communication tool, clinical adjunct, advocacy tool and a useful OHRQoL index. However, it is needed to be supported end-users’ feedback.

## Introduction

Sports and vigorous recreational activities are generally considered effective ways of improving physical fitness and health. However, Oro-Dental Trauma (ODT) during sports is unavoidable, particularly among contact sports players. It impairs Oral Health-Related Quality of Life (OHRQoL), especially in adolescents, which should be seriously considered. Traumatic dental injuries strongly influence adolescents’ physical, emotional and psychological well-being [1–4]; ODT causes detrimental impacts on social social-wellbeing [5–7] and incur high costs [8]. ODT in childhood may affect OHRQoL for the rest of life [1, 9]. Loss or fracture of an adolescent's anterior teeth has been found to have the most significant psychological impact on both parents and the adolescent, among all the other dental interruptions [1].

Dental trauma is a public health problem owing to its high frequency, severity and Impact on OHRQoL [10–12]. Moreover, in the context of Sri Lanka, there is a significantly high occurrence of oral & maxillofacial (OMF) injuries, including oro-dental trauma at schools and playgrounds, even amidst stringently imposed Covid-19 lockdown. This was revealed by the OMF injury surveillance data of the National Dental Hospital in Sri Lanka [13], indicating the magnitude of the problem when extrapolated to day-to-day life context without lockdowns, with unrestricted schooling and engagement in sports. However, there is a paucity of literature on adolescents' perceptions of their traumatised teeth and the impact it causes in their daily lives, especially in lower and lower-middle-income countries (LMIC).

Our literature review revealed scant literature on the impact of sports-related ODT, and no index was developed and validated for condition-specific oral-health-related-quality-of-life indices for oro-dental trauma to date. Studies have been conducted to assess the impact of dental trauma. However, they have utilised general OHRQoL instruments to assess the impact may be due to un-availability of a condition-specific tool.

Table 1 shows that the ODT-related impact has been assessed using general OHRQoL instruments. None of these instruments has included the factors of UDTN and the impact perceived after undergoing an oral healthcare system. Moreover, the ODT-specific index rather than the generic OHRQoL indices are more advantageous since they exclusively assess the symptoms and impacts of traumatic oro- dental injuries resulting in highly correlated and more sensitive outputs. Moreover, the ODT-specific OHRQoL tools can minimise the floor effect created by a generic index [14].

**Table 1:** Indices used to assess the dental trauma impact

	Research	Study population	The tool used and impact of dental trauma
I.	“Impact of traumatic injuries to the permanent teeth on the oral health-related quality of life in 12–14-year-old children” in 2002- by Maria Ilma de Souza Cortes, Wagner Marcenes, Aubrey Sheiham [15]	Brazilian schoolchildren aged 12–14 years	Oral Impact on Daily Performances (OIDP)
II.	“The impact of treatment of dental trauma on the quality of life of adolescents – a case–control study in southern Brazil” in 2007 by Maria Letícia Ramos-Jorge, Vera Lúcia Bosco, Marco Aurélio Peres, Ana Cristina Gerent Petry Nunes [16]	Hospital-based study in Brazil- adolescents aged 11 to 17 years	Oral Impact on Daily Performances (OIDP)
III.	"The unmet treatment need of traumatised anterior teeth in selected secondary school children in Ibadan, Nigeria" in 2010 by Mojirade Deborah Ajayi, Obafunke Denloye, Funmilayo Abiodun Solanke [17]	Nigerian schoolchildren aged 12–19 years	No
IV.	Quality of life impacts following childhood dento-alveolar trauma in 2010 by Jenny Marie Porritt, Helen Dawn Rodd, Sarah Ruth Baker [18]	Hospital-based study in the UK- children aged 7 to 17 years	Paediatric Quality of Life Inventory
V.	Impact of traumatic dental injuries with unmet treatment need on daily life among Albanian adolescents: a case–control study in 2011 by Dorina Sula Thelen, Tordis A. Trovik, Asgeir Bårdsen [19]	Albanian School children aged 16–19 year-olds	Oral Impact on Daily Performances (OIDP)
VI.	“Traumatic dental injury with treatment needs negatively affects the quality of life of Brazilian schoolchildren” in 2013 by Nailê Damé-Teixeira, Luana S. Alves, Thiago M. Ardenghi, Cristiano Susin, Marisa Maltz [20]	Brazilian schoolchildren aged 12 years	Child Perceptions Questionnaire (CPQ11-14)
VII.	“Oral health‐related quality of life and traumatic dental injuries in Brazilian adolescents” in 2014 by C. B. Bendo, S. M. Paiva, J. W. Varni and M. P. Vale [21]	Brazilian schoolchildren aged 8 to 10 years	Child Perceptions Questionnaire (CPQ8-10)
VIII.	Impact of dental trauma on Quality of Life among 11–14 years schoolchildren in 2017 by I. H. El-Kalla, H. M. Shalan and R. A. Bakr [4]	Egyptian schoolchildren aged 11 to 14 years	Child Perceptions Questionnaire (CPQ11-14)
IX.	“Traumatic dental injury and oral health–related quality of life among 15- to 19-year-old adolescents from Santa Maria, Brazil” in 2021 by L. D. Comim, Â. Dalla Nora, J. K. Knorst, D. N. d. O. Racki, J. E. d. A. Zenkner and L. S. Alves [2]	Population-based study 19 years old adolescents	Oral Health Impact Profile-14 (OHIP-14)
X.	“Impact of traumatic injuries to the permanent teeth on the oral health-related quality of life in 12–14-year-old children” in 2002- by Maria Ilma de Souza Cortes, Wagner Marcenes, Aubrey Sheiham [15]	Brazilian schoolchildren aged 12–14 years	Oral Impact on Daily Performances (OIDP)
XI.	“The impact of treatment of dental trauma on the quality of life of adolescents – a case–control study in southern Brazil” in 2007 by Maria Letícia Ramos-Jorge, Vera Lúcia Bosco, Marco Aurélio Peres, Ana Cristina Gerent Petry Nunes [16]	Hospital-based study in Brazil- adolescents aged 11 to 17 years	Oral Impact on Daily Performances (OIDP)

Utilising generic OHRQoL indices to assess ODT's perceived impact may lead to gross underestimation of the condition. One recent systematic review and a meta-analysis reported that uncomplicated traumatic dental injuries do not have a negative impact on the OHRQoL of children and adolescents [22]. None of the studies included had used ODT-specific valid and reliable tools but generic OHRQoL tools. Nevertheless, the authors have recommended further studies.

However, the South-East Asian region-related literature, including Sri Lanka, mainly discusses the prevalence of ODT and its risk factors [13, 23–25], reflecting that the perceived impact has been given scant attention. On top of that, ODT shows an increasing trend in the region [13, 26]. Our study is the first in South East Asia to develop an instrument and assess the Impact of ODT using a robust methodology. It can provide first-hand evidence on bridging this knowledge gap.

Several recent studies have reported that the contact sports-related oro-dental trauma prevalence is approximately 30% [27, 28]. Studies done among school children have revealed that contact sports such as boxing [29], martial arts [30], rugby [31], and basketball [32] bear a high risk for sports-related general injuries [33, 34]. Contact sports can potentially transpire dento-facial or temporomandibular injuries [27, 28]. They can cause facial [28] and cervical bone fractures [35] and brain concussions [36]. There has been an increase in traumatic accidents in sports throughout the previous decades [37]. The adolescent age group perceive and handles any trauma differently than young children and adults [38]. They can get deeply moved and manifest strong emotions like sadness, anger, frustration, and stress due to traumatised bodily injury, especially in the facial area [39].

It is important to appreciate that many authors have shown a significant association between TDI and OHRQoL notwithstanding the adolescents’ socio-demographic factors such as gender, age and skin colour and oral health factors such as caries prevalence, malocclusion and gingivitis [2, 20, 40].

The primary goal of oral health care professionals is to endorse OHRQoL and better patient-centred care. An index quantifying the impact of ODT improves patient-centred communication and is a pivotal adjunct for treatment planning and clinical evaluation. Understanding the impact of sports-related ODT will enable the development of effective strategies for impact mitigation, prevention, and advancement in sports dentistry. The ODT- impact assessment index can be used as an advocacy tool to enlighten the substantial burden of the prevalence and impact of ODT to the health programme planners and the contact sports and education stakeholders. It could be used as an advocacy tool for investing more in ODT preventive logistics and strategies. Further to this, recognising the impact of ODT-related detrimental effects on the OHRQoL of adolescents is essential to oral healthcare governing bodies to cater for training and logistic supplementation to oral healthcare professionals to provide the most contemporary preventive, treatment and rehabilitative techniques.

## Methods

**Fig. 1:**
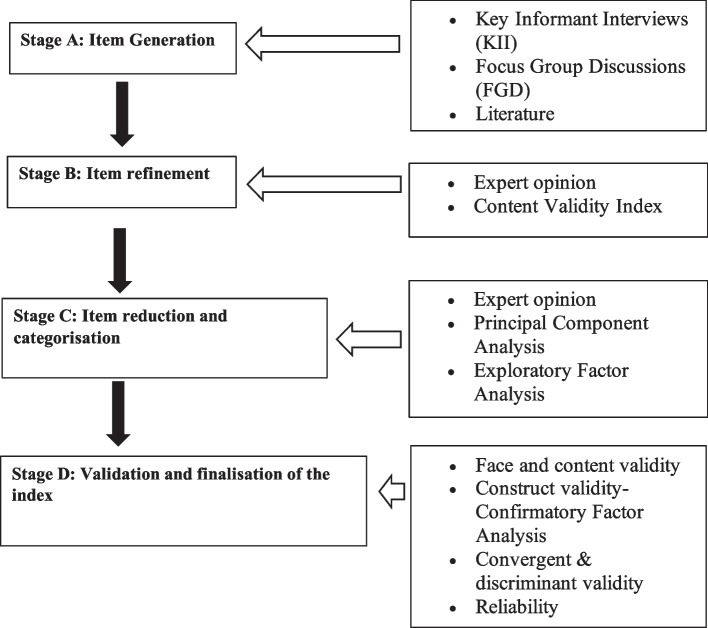
Conceptual framework of the development and validation of the AODTII

In this study, we developed and validated the AODTII as a condition-specific OHRQoL instrument targeting adolescents. AODTII is a 12-item composite index validated for Sinhala-speaking adolescents engaging in contact sports, and it subjectively evaluates ODT's impact on adolescents' quality of life. To accomplish this, we carried out a four-stage development and validation process, as illustrated in Fig. 1.

### Stage A: item generation

The AODTII was developed by generating a comprehensive list of items relevant to assess ODT's impact on adolescents' quality of life. The relevant items were formulated using several methods, such as conducting Key Informant Interviews (KII) and Focus Group Discussions (FGD). These approaches allowed us to gather inputs from experts in sports dentistry and other relevant disciplines, as well as from adolescents themselves. In addition to these qualitative methods, we also reviewed the existing literature on OHRQoL, which provided us with a conceptual framework to guide the development of the AODTII.

KII were conducted with a panel of experts (*n* = 10) consisting of specialists in public health, dental public health, restorative and prosthodontics, a psychiatrist, medical officers in sports medicine, and sports master in-charges/coaches. The characteristics of the expert panel are shown in Table 2.

**Table 2:** Characteristics of the expert panel contributed to the development of AODTII

	Speciality	Gender	Years of experience
I.	Specialists in public health	Female	22
II.	Specialist in dental public health	Male	31
III.	Specialist in Restorative and prosthodontics	Male	17
IV.	Specialist in Restorative Dentistry	Female	15
V.	Specialist in Restorative Dentistry	Female	31
VI.	Psychiatrist	Male	20
VII.	Medical officer in sports medicine	Male	11
VIII.	Sports master in-charge	Male	14
IX.	Dental surgeon	Female	15
X.	Dental surgeon	Male	13

Among the expert panel members explained in Table 2, the dental specialists were attached to the National Dental Hospital of Sri Lanka and the Institute of Oral Health, Maharagama, which are the main dental hospitals in the Colombo district. They are in charge of specialised units, and together with the dental surgeons, they manage dental trauma patients daily. Public health specialists and Consultant psychiatrists were attached to the public sector special programmes and were well-experienced with adolescents’ health issues. The medical officer in sports medicine was well-skilled in diagnosing, treating, and helping prevent injuries that occur during sporting events. The sports master in charge was engaging in athletic training and physical activities with good skills in communication with adolescents.

Four FGDs were carried out with adolescents who had had oro- dental traumatic injuries, and for this group, school-going contact sports players were enrolled. Each FGD was participated by ten adolescents. The FGDs were carried out until the saturation of the information. KII s and FGDs were performed according to pre-prepared guides.

The existing instruments regarding OHRQoL questionnaires scrutinised were the Oral Health Impact Profile-14 – validated Sinhala translation [41]; Oral Impact on Daily Performance (ODIP)- validated Sinhala translation [42]; Oral Health Impact Profile (OHIP) [43]; The Social Impact of Dental Diseases (SIDD) [44]; Geriatric Oral Health Assessment Index. (GOHAI) [45]; Dental Impact Profile (DIP) [46]; Dental Health Questions from Rand Health Insurance Company [47]; Subjective Oral Health Status Indicators [48]; Oral Health Quality of Life [49]; Dental Impact on Daily Living (DIDL) [50]; and Oral Impact on Daily Performances [51]. These instruments were assessed with their psychometric properties and used as one of the methods to identify the latent variables for AODTII. Moreover, theoretical aspects in the Practical guide for developing and using the scales measuring health [52] and research findings were also explored. At the end of the item generation step, an initial list of 43 items was created.

#### Stage B: item refinement

Personal interviews with experts were conducted to select and refine the most appropriate items from the initial list of 43. The same panel of experts who contributed to item generation were incorporated for the personal interviews. The items were assessed for relevance, appropriateness, and acceptability in the local context with addition, deletion, and modification items. The initial list of items was refined into 28 items with the expert opinion. Their responses were re-checked, reliability was assessed, and a summary report was shared to get feedback to minimise errors.

Then, the selected 28 items were assessed for their content validity using the Content Validity Ratio (CVR) Method developed by Lawshe [53]. The experts were given an explicit description of domains and a list of items. They rated each item on a four-point scale; 4 = Highly relevant, 3 = Quite relevant but needs rewording, 2 = Somewhat relevant and 1 = Not relevant to assess the content validity of the items. The expert opinion and consensus were obtained through the modified Delphi technique to finalise the draft of AODTII with identified constructs. Finally, 28 refined latent variables were selected as the draft AODTII to proceed with item reduction and categorisation.

The list of refined 28 items is listed below. The variables are numbered from Q1 to Q28, and we assigned that number to identify them in the development and validation stages.

##### Refined item list (*n* = 28)

- Q1_Uncomfortable while eating.
- Q2_Injuries to mouth/teeth cause problems with communication.
- Q3_Injuries to mouth/teeth cause problems with loud reading.
- Q4_Injuries to mouth/teeth cause hesitation in smiling.
- Q5_Get teased by friends due to injuries to mouth/teeth.
- Q6_Injuries to mouth/teeth cause dealing with the opposite sex with hesitation.
- Q7_Injuries to mouth/teeth cause hesitation to communicate with the teachers.
- Q8_Injuries to mouth/teeth make me uneasy with family members.
- Q9_Injuries to mouth/teeth make me uneasy among outsiders.
- Q10_Injuries to mouth and teeth cause nervousness in participating in gatherings.
- Q11_Feel reluctant to socialise with others due to mouth/teeth injuries.
- Q12_Untreated mouth/ teeth injuries are a significant issue in life.
- Q13_I can neglect my mouth/teeth injuries.
- Q14_Stressed for not taking treatments for mouth/teeth injuries.
- Q15_Poor self-image due to mouth/teeth injuries.
- Q16_Lack of time to repair teeth makes me suffer.
- Q17_Financial difficulties in repairing teeth make me suffer.
- Q18_Teeth are always at risk when engaging in contact sports; therefore, no point in repairing until sports are given up.
- Q19_Fond of having a broken tooth since it shows that he/ she is a tough boy/ girl.
- Q20_Filling colour is not satisfactory.
- Q21_Not satisfied with the quality of treatments received.
- Q22_Scared that the teeth will get damaged again.
- Q23_Reluctant to participate in sports again in case trauma occurs again.
- Q24_I am concerned about others' nasty remarks about my broken tooth/teeth.
- Q25_Problem due to pain.
- Q26_Problem due to sensitivity.
- Q27_Pain was problematic even after restoring the tooth.
- Q28_Not satisfied after the dental treatments.

##### Translation of the draft index

Translation of the index and scales into the Sinhala language was carried out since it was the most used language within the catchment area where the data collection was carried out. The conceptual equivalence, item equivalence, semantic equivalence, operational equivalence and measurement equivalence between the original and translated versions were considered in this process. A professional translator translated the refined item list into the Sinhala language, and an independent translator, blind to the translated version, performed the back-translation. Finally, the discrepancies were sought out.

Despite being a valid and reliable instrument, it should be feasible to administer with a manageable number of valid items [53]. We applied qualitative validation, content validity index, and Principal Component Analysis (PCA) to achieve this. The exploratory Factor Analysis (EFA) method was used to determine the underlying constructs to manifest the factor structure of the short-listed and refined variables [52].

Prior to the PCA and EFA, the draft of the AODTII was pre-tested among a feasible sample of 20 adolescents with ODT (From the DS Senanayake College, Colombo 7) to ensure clarity and the ability to understand the subjects of the items. Pre-test findings led to identifying the grey areas, which were modified further.

The sample size for PCA analysis and EFA was determined according to the rule of thumb that there should be five subjects for each variable or at least 100 subjects [52]. Since there were 28 latent variables available for PCA, 140 subjects were assigned (five subjects per one latent variable) for the EFA. The sample recruited comprised 140 adolescent contact sports players aged 13 to 18 who had traumatic dental injury/injuries, either: uncomplicated/ complicated dental trauma, contusions, or any soft tissue lacerations. EFA data was collected at Siri Piyarathana College, Padukka, DS Senanayake College, Colombo 7, Rajasinghe Central College, Hanwella and Central College, Homagama, which are randomly selected schools from Colombo District.

The items were reduced by the PCA method. First, the selected 28 variables were assessed for their factorability in a correlation matrix. The correlation coefficient > 0.20 was used to assess the factorability inspection of the correlation matrix [54]. Then, initial factors were extracted via PCA, identifying the number of variables to retain. Eventually, an item list with 12 was refined and retained as the final list for the AODTII (Q1_Uncomfortable while eating, Q4_Injuries to mouth/teeth cause hesitation in smiling, Q5_Get teased by friends due to injuries to mouth/teeth, Q10_Injuries to mouth and teeth cause nervous on participating gatherings., Q11_Feel reluctant to socialise with others due to mouth/teeth injuries, Q13_I can neglect my mouth/teeth injuries, Q14_Stressed for not taking treatments for mouth/teeth injuries, Q15_Poor self-image due to mouth/teeth injuries, Q20_Filling colour is not satisfactory, Q21_ Not satisfied with the quality of treatment received, Q25_Problem due to pain, and Q26_Problem due to sensitivity).

According to Strainer and Norman [52], it was exploratory when there was no predefined impression of the construct or the number of dimensions in a set of variables. Thus, we performed EFA with the list of 12 items to identify the underlined factors, screening variables, sampling variables and clustering of subjects. Underlined factors are homogenous variable clusters reflecting the characteristics of the specific domain, and screening variables helped to select the underlying constructs.

#### Stage C: item reduction and categorisation

Factor analysis assessed whether the items in a multi-dimensional scale were assigned to the correct subscales using their factor loadings [52].

We used EFA to select the model. Promax rotation was performed to examine whether a variable was related to more than one factor. It is an oblique rotation that assumes correlations among the identified latent factors. Studies assessing the psychological constructs contain variables that are co-related to each other [52]. Therefore, Promax rotation was well-fitted for this study. The rotation has been repeated until a satisfactory model is built with four factors. We named the four constructs per the impact denoted by the underlying variables. The factors are denoted by ‘F1, F2, F3 and F4’.

1. F1- Social impact mingled with peer pressure (Q10, Q11, Q4, Q5)
2. F2- Physical impact (Q26, Q25, Q1)
3. F3- Impact due to oral healthcare system (Q20. Q21)
4. F4- Impact due to unmet dental treatment need (Q15, Q14, Q13)

#### Stage D: validation and finalisation of the AODTII

##### Face and content validity

The judgemental validity of AODTII was appraised with face and content validity. The latent variables of the AODTII were assessed for face validity by the same panel of experts. The experts assessed content validity with the percentage of agreement for each variable by calculating a content validity ratio.

##### Construct validity

Construct validity of the Sinhala version of the AODTII was appraised by Confirmatory Factor Analysis (CFA) on a separate study sample from Piliyandala Central College and Henry Olcott Maha Vidyalaya, and St Johns College, Nugegoda. We recruited 132 adolescent contact sports players with uncomplicated/ complicated ODT or any soft tissue injury to the oro- dental area for CFA.

The software for CFA was IBM SPSS Amos Version 27. The RML (Robust Maximum Likelihood method) was used to estimate the model parameters.

The sample consisted of 132 participants. Therefore, it was amply adequate for CFA (eleven participants per variable). The sample adequacy was verified with the KMO (Kaiser–Meyer–Olkin) and Bartlette's test. The resulting KMO value of 0.734 indicated an adequate sample size since it exceeds the cut-off value of 0.6. Moreover, the higher Chi-square value and the significance value less than the referral value of 0.05 further reinforced the sampling adequacy. The sample was assured with multivariate normality, no outliers, and no multicollinearity.

First, we developed a two-factor model with AMOS. The chi-square value was 216.44, and the RMSEA (Root Mean Square Estimate of Approximation) value was 0.146. Although this model showed a 'somewhat good fit, we tried a better model with three factors.

Then we tried a Three -factor model by keeping the social/ peer pressure and the physical impact domains together while disintegrating the impact due to the health system domain and the domain of acceptance of Unmet Dental Treatment Needs (UDTN). The chi-square value was 196.52, and the RMSEA value was 0.140, which showed a slight improvement but did not fit into an acceptable 'good' model.

Finally, a four-factor model was constructed, and the Social/peer pressure domain was also disintegrated from the physical impact domain. The chi-square value was 169.78, and the RMSEA (Root Mean Square Estimate of Approximation) value was 0.082, which manifested a much better improvement fitting into an acceptable model.

##### Convergent and discriminant validity

The construct validity of a scale was ensured by convergent and discriminant validity. Convergent validity evaluates the fact that the items that should be related are related. In contrast, the discriminant validity ensures that unrelated items are kept unrelatedly [52]. The convergent and discriminant validity was assessed in a multi-trait scaling analysis.

##### Reliability

Cronbach's alpha was measured to test the internal consistency. The reliability of the AODTII was assessed by the internal consistency of the individual subscales and the 'test – re-test'' method. Test–retest reliability was assessed on a group of 20 children after two weeks of initial administration of the AODTII. The median scores obtained from all subscales were compared. All subscales manifested significant and high correlations.

#### The cut-off values of the AODTII

The methodology used to calculate the cut-off values for AODTII is illustrated below.

I The final factor matrix in the EFA dataset was used, where each participant's response for the selected 12 questions was obtained on the four-point Likert scale.
II The weighted score for each factor was calculated separately for each participant. Each variable’s factor loading in PCA was used to calculate the weighted score for each factor.
III Then, the weighted composite score was calculated by summating all four weighted scores.
IV Composite scores were divided into four quartiles, and the Q1 (25^th^ percentile value), Q2 (50^th^ percentile value) and Q3 (75^th^ percentile value) were obtained.
V In the weighted composite score of AODTII, Q1=21.12, Q2=24.76 and Q3=28.21. The cut-off values of AODTII were assigned according to their position in the quartiles. The cut-off values demonstrate the Impact of ODT perceived by adolescents, as shown in Table 3.

**Table 3:** Cut-off values for AODTII

	**Composite score**	**Impact**
(i)	Less than 21.12≈ ^a^ 21	Less than significant
(ii)	Between 21.12 and 28.21,≈ 21 and 28	Significant
(iii)	More than 28.21≈ 28	Highly significant

^a^ Approximately equal

**Fig. 2:**
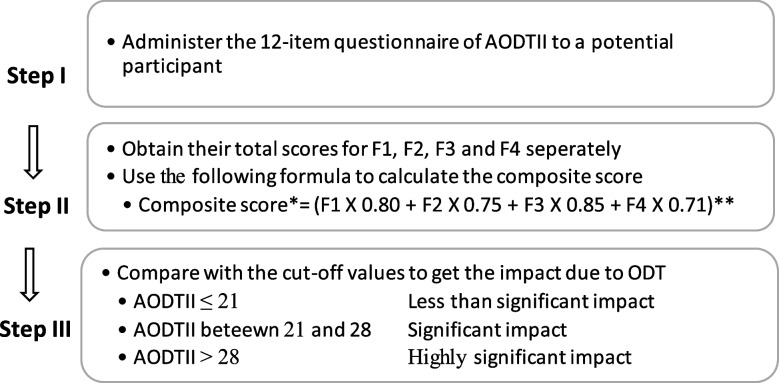
Assessment of the AODTII on a potential participant. *Composite score denotes the AODTII value. **Average factor loading for each was derived from the PCA

AODTII is simple and feasible to calculate by the end users. It can be assessed in three steps. It is illustrated in Fig. 2.

Moreover, for simplicity, AODTII more than 8 (as per the Q2 value = 24.76 ≈ 25 can be considered as the dichotomised cut-off to differentiate participants for having a less or substantial impact. However, we recommend assessing AODTII using the cut-off values illustrated in Fig. 2.

## Results

### Item generation

Fourteen personal interviews and four focus group discussions were considered adequate samples for qualitative analysis, as the 12th interview reached data saturation. There was a strong level of agreement between the coders for qualitative analysis (weighted kappa agreement 0.7). A list of 32 items was generated with the personal interviews and the FGDs.

Nine items were generated from reviewing the existing instruments, and two from reviewing the theory and research findings. The total number of items in the initial list was 43.

### Item refinement

Item refinement was carried out with the expert opinion of the key informants using the modified Delphi technique, following the Content Validity Ratio method developed by Lawshe [53].

The following formula was used to assess the CVR.

CVR = (n_e—_N/2) /(N/2) (n_e_ = the number of experts who agreed; *N* = total number of experts).

CVR ranges between -1 to + 1. As we had ten experts, the items with lower values than 0.62 were discarded according to the Lawshe Table [53].

The item refinement process ended up resulting in 28 items.

### Item reduction and categorisation

#### Principal component analysis and exploratory factor analysis

The sample for PCA and EFA consisted of 13 to 18 years old adolescents with traumatic dental injury or soft tissue lacerations in the oral tissues. Out of the sample, 57.9% were less than 15 years, while 69% were males.

Twenty-eight latent variables were retained for the PCA, and the responses were recorded on a four-point Likert Scale. Each item varied from one to four and gave an aggregate score of 28 to 112. Eyeballing of the histograms of all 28 items revealed the normal distribution of most of the items. Since the PCA and EFA are based on the correlation between variables, the linearity of data was assessed on a bivariate correlation matrix. A random sample of bi-variate scatter plots was examined, and observed the linear relationship.

EFA is sensitive to outlying cases, and therefore the item values were converted into standardised scores (Z-scores), and no value was identified ± 3.29. Thus, no univariate outliers were observed.

KMO is an index for comparing the magnitude of the observed correlation coefficients with the magnitude of the partial correlation coefficients. KMO values closer to one indicate sizable sampling adequacy (0.8 or higher as highly acceptable; 0.7 as acceptable; 0.6 as average; and less than 0.5 as unacceptable). KMO value of 0.767 indicated the sizable sampling adequacy since it was well above the value of 0.6. Bartlett's test of sphericity is used to test the null hypothesis that variables in the sample correlation matrix are not correlated [36]. Since Bartlett's test value gave a *p*-value less than 0.000, it indicated a significant value. Thus, the sample was well adequate for PCA and EFA.

The correlation matrix values were over the value of 0.2, and the majority were around 0.3. Thus, an excellent inter-item correlation was there for EFA. The determinant showed a value of 0.0258, well above the referral value of 0.00001; therefore, this correlation matrix was positive for factor analysis. There were no 'very high' co-relations, such as 0.8 or above, in which case it was present, indicating a 'red flag' of multicollinearity. As all the correlations were close to 0.3, denoted co-relations without multicollinearity. Communalities explain the proportion of the variability of the identified variables. The minimum value was 0.463, all other values were above 0.530, and the maximum was 0.777. This suggested that the values were well fit for PCA. Therefore, all 28 factors were considered in PCA.

The rotation of the variables was carried out using various oblique rotational methods. Oblimin, Promax and quatrimax rotational methods were performed. Finally, a good model fit to the data was obtained with Promax rotation, with the scores set to 0.4. Then, variables were removed and added until a sound factor matrix was obtained. The features that were looked for were the factor matrix that loaded the variables cleanly under a specific construct. The minimum number of latent variables with higher factor loading was obtained as the best matrix.

The communalities of the retained 12-factor matrix are given in Table 4. (The variable numbers are given as they appeared in the entire 28-item list).

**Table 4:** The communalities of the factor matrix

Variable	Extraction
Q1_**Uncomfortable** while **eating**	0.431
Q25_Problem due to** pain**	0.786
Q26_Problem due to **sensitivity**	0.831
Q4_Injuries to mouth/teeth cause **hesitation in smiling**	0.649
Q5_Get **teased by friends** due to injuries to mouth/teeth	0.601
Q10_Injuries to mouth and teeth cause **nervousness in participating gatherings**	0.703
Q11_Feel **reluctant to socialise with others** due to mouth/teeth injuries	0.735
Q13_**I can neglect** my mouth/teeth injuries	0.484
Q14_**Stressed** for not taking treatments for mouth/teeth injuries	0.650
Q15_**Poor self-image** due to mouth/teeth injuries	0.646
Q20_Filling **colour is not satisfactory**	0.737
Q21_**Not satisfied with the quality** of treatment received	0.699

The factorability of data was assessed on a matrix of communalities. The communalities ranged from zero to one. Higher values in the matrix indicated better factorability. In the communality matrix, all the values were above the cut-off value of 0.3. The minimum value was 0.431, and the maximum was 0.831, which indicated a good level of factorability.

Items grouped were based on the Eigenvalues. Factors with an Eigenvalue above one were considered relevant. This is explained in Table 5.

**Table 5:** Loading of factors according to their Eigenvalue

Component	Eigenvalues	Total Variance %	Cumulative %
**1**	3.639	30.323	30.323
**2**	1.551	12.923	43.246
**3**	1.497	12.472	55.718
**4**	1.266	10.551	66.270
5	0.768	6.399	72.669
6	0.718	5.987	78.656
7	0.617	5.140	83.796
8	0.548	4.566	88.363
9	0.490	4.085	92.448
10	0.396	3.301	95.749
11	0.291	2.425	98.174
12	0.219	1.826	100.000

During factor extraction, 66.27% of the variance was explained by the factor structure, which exceeds the Eigenvalue of one. Eigenvalues of the extracted latent factors ranged from 1.266 to 3.639.

Four latent factors were identified by EFA, followed by Promax with Keiser Normalization rotational method. The scree plot identified the four factors above the Eigenvalue of one. The Spearman correlation coefficient among the identified factors was assessed on a component matrix. All the values were below 0.5, indicating a good level of correlation without any highly correlated values such as 0.7, 0.8 or above. The final factor matrix is shown in Table 6.

**Table 6:** The final factor matrix in principal component analysis

Variable	Component			
	**F1**	**F2**	**F3**	**F4**
Q10_Nervous to participate in gatherings	0.873			
Q11_Reluctant to socialise	0.852			
Q4_Hesitation for smiling	0.751			
Q5_Get teased by friends	0.717			
Q26_Sensitivity- problematic		0.929		
Q25_Pain -problematic		0.884		
Q1_Uncomfortable while eating		0.444		
Q20_Filling colour not satisfactory			0.877	
Q21_Not satisfied with the quality of treatments			0.830	
Q15_Poor self-image				0.771
Q14_Stressed for not taking treatments				0.766
Q13_Can neglect				0.592

Thus, dimension reduction was performed using PCA and categorisation into four factors by the EFA. Statistical analysis was performed in SPSS version 29. It was interesting to note that all the variables had a loading above 0.4 without cross-loadings. The pattern matrix with the identified 12 variables under four factors is shown in Table 6. Moreover, the identified factors are defined as follows.

- **F1**- Social impact mingled with peer pressure- Impact perceived by adolescents due to oro- -dental trauma while socialising. The impact of peer pressure from their peer group is emphasised here. (All the factors loaded more than 0.7).
- **F2-** Physical impact of trauma- Impact perceived by the adolescent due to the 'clear-cut.' physical effects such as pain and sensitivity experienced (Two factors loaded more than 0.8 and one with 0.4).
- **F3-** Impact caused by the oral health system- Impact perceived by the adolescent regarding the quality of treatment they receive from the oral health care delivery system. (All the factors loaded more than 0.8).
- **F4-** Impact due to unmet dental treatment need- The impact perceived by the adolescent related to their image and whether they are concerned about the oro- dental traumatic issues (All the factors loaded more than 0.7 except one with 0.5).

### Psychometric properties of AODTII

#### Construct validity

**Fig. 3:**
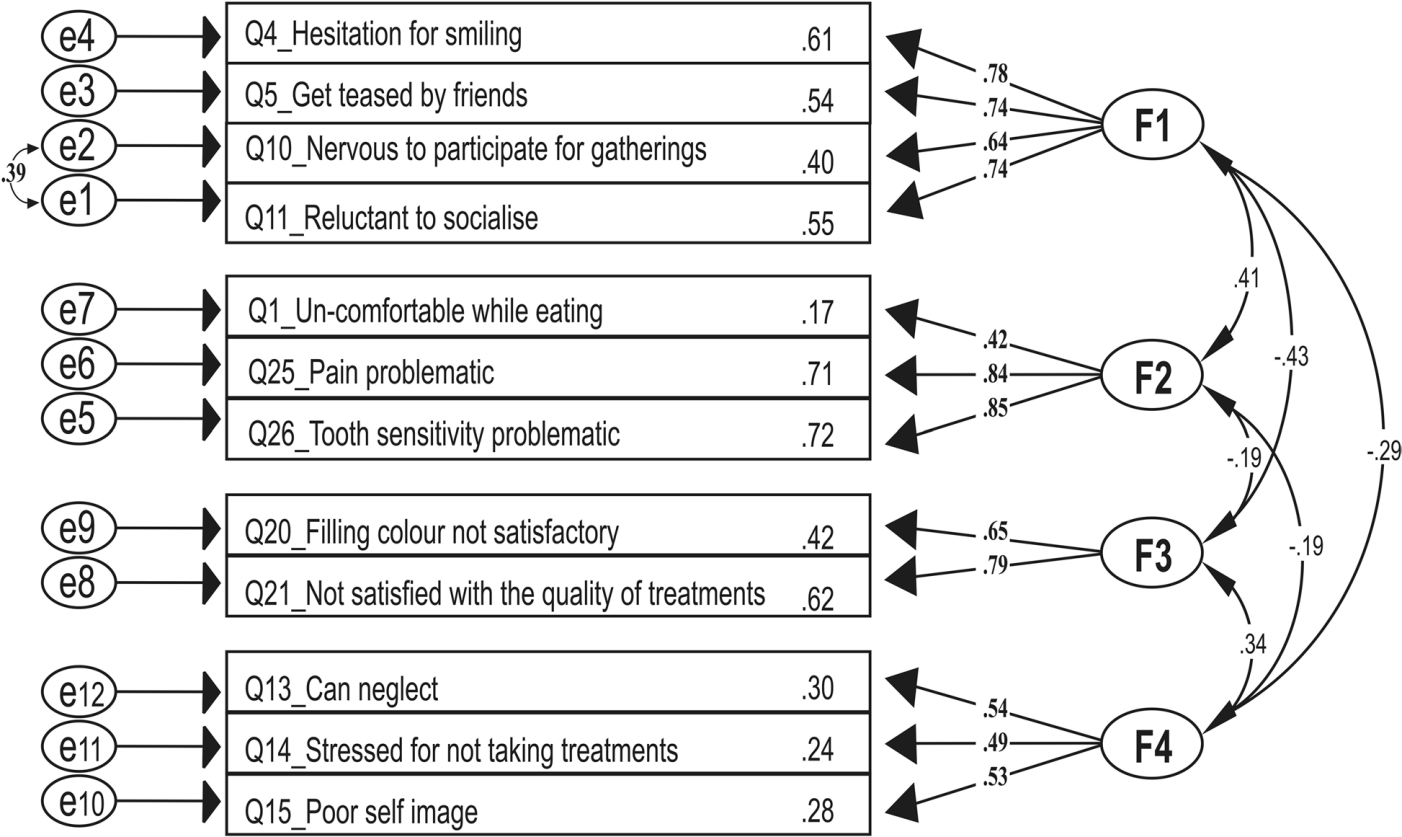
Structure equation model with four factors

The normality assessment verified the CFA dataset's suitability for Structural Equation Modelling. The measure of skewness for each item indicated the absolute value, ranging from -0.727 to 0.389 depicting normality. It was further assured as the kurtosis was within the range of (-1.642 to 4.040) in all the items, well within the normal range of -10 to 10. Moreover, the dataset was checked for outliers by checking the observations farthest from the centroid (Mahalanobis distance). The dataset was without outliers, as the p1 and p2 values for each item were more than 0.001 [52]. Figure 3 shows the Structural equation model for AODTII.

CFA is an extension of EFA to test the construct validity of the index developed. The structural equation model extends path analysis and Fig. 3 shows how the latent variables are related.

The model fit statistics are given in Tables 7 and 8.

**Table 7:** Absolute model fit indices of the AODTII obtained by confirmatory factor analysis

X^2^	df	p	RMSEA	GFI	AGFI	SRMR
66.33	47	0.033	0.054	0.931	0.885	0.070

*RMSEA* Root Mean Square Estimate of Approximation (< 0.08 desired), *GFI* Goodness of Fit Index: (> 0.90 desired), *AGFI* Adjusted Goodness of Fit Index: (> 0.90 desired), *SRMR* Standardised Root Mean Square Residual (< 0.05 desired)

**Table 8:** Relative and parsimony model fit indices of the AODTII obtained by confirmatory factor analysis

Relative fit indices		Parsimony fit indices	
**CFI**	**NFI**	**PGFI**	**PNFI**
0.957	0.871	0.561	0.620

*CFI* Comparative Fit Index (> 0.95 desired), *NFI* Non-Normal Fit Index (> 0.95 desired), *PGFI* Parsimony Goodness of Fit Index: (> 0.05 desired), *PNFI* Parsimony Normed Fit Index (> 0.5 desired)

The absolute model fit indices resulted with good fit as shown in Table 7 and the Relative and Parsimony fit indices were satisfactory as shown in Table 8.

### Convergent and discriminant validity

Strong correlations were observed among the items in a particular sub-scale, ensuring convergent validity. The items in different subscales were weakly correlated, ensuring their discriminant validity. Accordingly, the scale's good convergent and discriminant validity further amplified the construct validity. The multi-trait scaling is shown in Table 9.

**Table 9:** Multi-trait scaling analysis of AODTII (*N* = 132)

Variable	Component			
	**FI**	**FII**	**FIII**	**FIV**
Q10_Nervous to participate in gatherings	**0.857**	0.255	0.256	0.175
Q11_Reluctant to socialise	**0.831**	0.157	0.164	0.161
Q4_Hesitation for smiling	**0.791**	0.334	0.312	0.210
Q5_Get teased by friends	**0.766**	0.303	0.309	0.190
Q26_Tooth sensitivity problematic	0.224	**0.908**	0.125	0.116
Q25_Pain problematic	0.290	**0.879**	0.127	0.320
Q1_Uncomfortable while eating	0.340	**0.552**	0.401	0.307
Q20_Filling colour not satisfactory	0.214	0.107	**0.854**	0.174
Q21_Not satisfied with the quality of treatments	0.292	0.177	**0.833**	0.113
Q15_Poor self-image	0.120	0.276	0.109	**0.756**
Q14_Stressed for not taking treatments	0.258	0.231	0.113	**0.740**
Q13_Can neglect	0.167	0.243	0.391	**0.639**

### Reliability

Appraising the reliability of the AODTII was done by calculating the internal consistency and test–retest reliability using data from the validation study. All subscales had Cronbach's alpha value of more than 0.7. Cronbach's alpha was significant at the *p*-value < 0.01 (2-tailed). All domains of the AODTII exceeded the criteria of 0.7, manifesting a satisfactory internal consistency [55]. A high level of internal consistency within all subdomains implied that the AODTII had an acceptable reliability level.

The corrected item-total correlation coefficient matrix shown in Table 10 was used to assess the homogeneity of the scale. The Cronbach alpha of the 12-item scale was 0.768. The correlation coefficient of the individual item with the entire item list was assessed. The resultant correlations were also compared with Cronbach's value if the item was eliminated.

**Table 10:** Number of items and cronbach’s alpha value in each domain for overall scale of AODTII (*N* = 132)

Sub Scales	No. of Items	Cronbach’s Alpha Value	95% Confidence Interval	
			**Lower Boundary**	**Upper Boundary**
I. Social/ peer pressure	04	0.766	0.693	0.825
II. Physical Impact	03	0.726	0.637	0.797
III. Impact due to oral healthcare system	02	0.776	0.648	0.768
IV. Acceptance of UDTN	03	0.712	0.657	0.786
**Total**	**12**	**0.768**	**0.630**	**0.921**

Table 11 explains the reliability analysis and the Cronbach’s alpha value for the total score was 0.768 (95% CI = 0.630- 0.921). All the alpha values, ‘if item deleted,’ was less than 0.768.

**Table 11:** Reliability analysis (*N* = 132)

AODTII Item	Corrected item-total correlation	Alpha, if the item deleted
Q1_Uncomfortable while eating	0.465	0.747
Q4_Hesitation for smiling	0.519	0.739
Q5_Get teased by friends	0.557	0.733
Q10_Nervous to participate in gatherings	0.500	0.740
Q11_Reluctant to socialise	0.451	0.747
Q13_Unhappy for neglection	0.250	0.767
Q14_Stressed for not taking treatments	0.266	0.765
Q15_Poor self-image	0.351	0.758
Q20_Filling colour not satisfactory	0.394	0.753
Q21_Not satisfied with the quality of the treatments	0.411	0.751
Q25_Pain problematic	0.342	0.759
Q26_Sensitivity problematic	0.363	0.756

The correlation coefficient of the corrected-item-total matrix evaluated the homogeneity of the AODTII. It ranged from 0.250 to 0.557, denoting all the values obtained a higher value than the cut-off of 0.2, which is the recommended value that an item is included in an instrument [52].

The test–retest method was applied to 20 participants at a two-week interval for individual domains, and the correlation coefficient values ranged from 0.711 to 0.978, with a total score of 0.832. The test–retest results are shown in Table 12. It further assured the reliability of the index.

**Table 12:** Test re-test reliability results of the AODTII (*N* = 20)

Dimensions and Subscales of AODTII	Intraclass Correlation	95% CI	df	Level of significance
Social/ peer pressure	0.938	0.893 – 0.963	168	*P* < 0 000
Physical impact	0.822	0.681 – 0.912	96	*P* < 0 000
Impact due to health sys	0.950	0.908—0.976	72	*P* < 0 000
Acceptance of UDTN	0.678	0.433—0.840	120	*P* < 0 000
Total scale	0.832	0.711 – 0.978	552	*P* < 0 000

All the sub-scales manifested a correlation coefficient value of more than 0.7 except the UDTN domain. However, that also achieved 0.678, and the AODTI was the reliable index.

A limitation of this study is that there is no assessment of the acceptability of the tool by the end-users other than the high participation rate (98%) of adolescents in the survey. Qualitative methods could have accomplished this by enrolling end users: dental surgeons, school teachers and adolescents engaging in contact sports. Data quality was ensured, as the principal investigator only collected data. Inter-rater agreement was calculated and resulted in good Intraclass Correlations.

### Details of data entry and analysis

Collection, data entry and analysing, has been carried out by the Principal Investigator. Data cleaning was done prior to data entry in the IBM SPSS software version 27 (Descriptive analysis and EFA) and 29 (Structural Equation Modelling). All the authors contributed in data analysis, interpretation and reporting.

### Ethics approval and consent to participate

The study received approval from the Ethics Review Committee, Faculty of Medicine, University of Colombo, Sri Lanka (EC-17–160). All procedures performed in the study involving participants were by the institution's ethical standards and with the 1964 Helsinki Declaration and its later amendments or comparable ethical standards. Written informed consent was obtained from each patient before participation in the study.

## Discussion

AODTII is a 12-item index developed and validated for assessing the perceived impact of untreated or treated contact sports-related oro-dental trauma among Sinhala-speaking adolescent school children. AODTII could be useful in assessing the perceived impact of oro-dental trauma among adolescents unrelated to sports. It could be used in clinical settings as a clinical adjunct as well as in community settings as a screening tool to detect unmet oro-dental trauma treatment need. Further, it could be useful in epidemiological studies on the burden of disease studies in oro-dental trauma among adolescents. Moreover, AODTII will be helpful in prioritising adolescent patients for restorative dental treatment in economic crises and resource constraints.

Development and validation of the novel tool was motivated by the literature review that revealed the scant instruments for ODT-related impact assessment. As the novel tool precisely assesses the Impact of ODT validly and reliably, only with 12 questions, it is feasible for the end-users and simple to be calculated the results by the administrators.

A mixed method approach has been used to develop this index, validated by a rigorous methodology comprising exploratory factor analysis, confirmatory factor analysis, and structural equation modelling. The reliability of the index was assessed by Cronbach's alpha and test–retest reliability. Results revealed the AODTII as a valid and reliable condition-specific oral-health-related quality of life (OHRQOL) index for adolescents. The novel index has good psychometric properties. Moreover, as the index has only 12 items, it is feasible in the application and can be self-administered.

Health-related quality of life (HRQoL) is a subjective construct, and it is multi-dimensional. It can be defined as "the perceived impact of health on an individual's potential to live a subjectively fulfilling life [52]. ODT in childhood and adolescence is recognised as a potential public health problem, given its prevalence and consequences [56] and significantly disturbs their OHRQoL. Thus, OHRQoL measures are essential in patient-centred care. It is well explained in the review on "measuring health-related quality of life" [56]; that the "physiologic measures provide much information to clinicians as two patients with the same condition may have different role functions and well-being. One may neglect the condition while the other may be depressed over it". The psychological impact assessment factor got the highest loadings in PCA at the development stage of AODTII. The psychological impact represented a more significant amount (more than 50%) of the cumulative variance explained by the four factors.

The assessment of the Impact concerning ODT is a new concept in Sri Lanka that deviated from conventional caries and oral cancer-related QoL scales. It can be used to initiate the arena of sports dentistry in Sri Lanka, which needs improvement in the context. AODTII can be used to advocate for authorities to provide ODT protective instruments to the school-contact sports players as an initial step. AODTII can be used as an oral health promotional tool where the coaches and adolescents contact sports players can be motivated to use protective gear. It can be used as a clinical adjunct in better communication with the patient and to triage ODT patients related to the perceived impact. However, the authors recommend further research by the end users at the oral healthcare provider regarding the validity of AODTII as a clinical adjunct.

Our study shows that the impact of dental trauma can be assessed with ADOTII with good validity and reliability. Child Perception Questionnaires (CPQ) _11 – 14_ is another OHRQoL instrument with 25 questions with good validity and reliability. It was developed and validated under oral symptoms, functional limitations, emotional well-being, and social well-being [57] and validated among children with caries, cleft lip, and palate. However, their focus was not on assessing the ODT burden of adolescents since they are a unique age group considering facial aesthetics and peer pressure vastly in contrast to older adults. Moreover, an instrument tailor-made for them should elicit their mindset's subtle emotional and attitudinal complexities. However, as AODTII has been finalised with only 12 items, it may be a limitation to demonstrate all the constructs precisely. However, unlike other OHRQoL instruments, it only assesses the impact related to ODT and overrides the limitation.

Some authors suggest that un-complicated dental trauma results in no significant impact on children and adolescents [22]. However, the tools used in the studies were not trauma-specific, and the authors also discuss that using a proxy measure such as the Early Childhood Oral Health Impact Scale (ECOHIS), where parents/ caregivers report the child's impact, would result in diluted results. Moreover, other authors show that adolescents treated for enamel and dentine trauma indicate poor quality of life than their counterparts [16].

AODTII has unique features in which UDTN and health system impact, if any, are also considered. Thus, it enlightens the real perceived impact of adolescents towards ODT. It has been reported that UDTN is a significant problem in LMICs such as Nigeria, and the elapsed time for treatment of TDI is as high as 3.5 years [17]. Another recent study among adolescent school children in Panchkula, India, has reported that anterior dental trauma is a significantly neglected condition related to oral health [58]. UDTN could be due to the availability of the services, accessibility and affordability. A study done in AODTII has captured this construct by ‘poor self-image, stressed for not taking treatments and can neglect’ scenarios as they have a negative impact reflected with ‘poor self-image’ or they are in dire need of treatment but restricted may be due to pre-mentioned factors or they can just neglect their ODT status. The total variance explained by UDTN is almost equal to that of physical impact. Thus, adolescents perceive it the same as the physical impact, which shows the importance of UDTN in the local context. Similar results were found among Albanian adolescents whose UDTN was associated with reduced OHRQoL assessed using the OIDP index [59].

It is evident in the literature that timely dental trauma management improves patient outcomes [60]. Assessment of “dental Patient Reported Outcomes (dPROs) following TDI is a novel research area where clinicians, as well as patients, are assisted in choosing the best management option(s) for each patient [61].” Moreover, our index can capture the negative impact of sub-standard treatment under the "impact due to health system'. Considering both treated and untreated ODT cases in AODTII is an advantage in assessing the construct of dPROs. It can be directly utilised in future research related to ODT.

Although patient-centred care is a controversial issue in lower and LMIC [62], Sri Lanka has achieved excellent health outcomes proportionate with its income [63], and evidence from Sri Lanka reveals that the factors related to clinic context, treatment process, convenience and outcome of care were well satisfied by the patients in an oral healthcare setting [64]. Against this backdrop, it is logical to recommend the introduction of indices such as AODTII in the context of better patient-centred care. However, this should be verified by further research. In our index, ‘Impact due to health system’ explained the least variance in the model; however, the construct is significant as its Eigenvalue was more than one. The variables retained in the final AODTII to assess the construct of the health system are only related to the colour of the dental restoration and the quality of treatment received. It is one of the constraints in the index.

### Methodological aspects

The appropriateness of the selected items on the scale was judged by a panel of experts on the face and content validity. Face validity indicated that the selected variables were assessing the desired qualities of the face of it. The panel of experts assessed content validity to verify whether the variables assessed all the relevant contents. These were assessed in three rounds with the same panel of experts and indicated a good level of validity. In the pattern matrix in PCA, the factor loading for the variable 'Q1_uncomfortable while eating' had the least value of 0.444, while almost all the others loaded more than 0.7. However, the experienced panel of experts decided unanimously to keep that variable in the model. In the final round, the CVR was 88.3, ensuring that the final matrix avoided any overinclusive poor and irrelevant items. Profound authors have advised this methodology [52, 53], which has been used in tool development procedures [43].

The consensual validity of the variables was well-established by reaching a consensus with multi-disciplinary experts in dentistry, psychiatry, and sports. The consultants in relevant fields, sporting coaches, experienced master in-charges (MIC -Sports) in schools, and dental surgeons contributed to the process. The literature shows this is the practical norm of consensual validation [65]. As gathering all the panel members in person was not feasible, we used the Modified Delphi technique to meet the consensus by e-mailing amended versions of the tool with content corrections and we compiled comments using individual reports. The final reports were e-mailed and obtained consensus.

PCA is a multivariate statistical approach commonly used in psychology, education, and, more recently, in health-related professions [66]. It is usually the first step in building scales and new matrices [67]. The pre-analysis process was carried out to ensure whether a stable population factor structure emerged from the sample, items were appropriately scaled, free from bias, and the data was appropriate for EFA [68]. The stable factor structure was ensured by obtaining well above the minimum sample size of 100 subjects (*n* = 140). According to Kline, 1986, the absolute minimum number of subjects ranged from 100 to 200 [69].

The sampling adequacy was further ensured by the KMO measure and Bartlett's test of sphericity. Some amount of skew and kurtosis with univariate normality is accepted for EFA [70]. According to the 'FA user's Gide', less than 25% of variables adversely affected by skew and kurtosis were acceptable. The rule of thumb was that between + 2 and -2 is acceptable for EFA [68]. The study sample had skewness and kurtosis in the range of 0.075 to 1.198 and was well accepted for EFA.

Many authors deem appropriate in the correlation matrix a necessary psychometric requirement [52, 66, 68–70]. All the items should correlate with each other with a value close to 0.3. The very high values denote multicollinearity, and significantly fewer values suggest redundancy of the variable. When observing the correlation matrix, it was evident that most variables correlated in the 0.441 – 0.298. Thus, the dataset was suitable for extraction, and the derived matrix was appropriate for FA.

Extraction of factors has been carried out, resulting in retaining the factors which are necessary to reproduce the initial correlation matrix adequately. The number of factors to be extracted depends on the Eigenvalue (Eigenvalue of more than one) and statistical method for computing pattern coefficients [48]. In the current study, the Eigenvalues of the factors ranged from 3.639 to 0.219. Four factors could be identified above the Eigenvalue one; the cumulative variance explained by those factors was 66.3%. The cumulative variance of more than 60% is sufficient for EFA [44]; however, future studies must research the variability these four factors do not explain.

The rotation method is selected according to the correlation expected among the variables. According to the literature, there are broadly two rotational methods: orthogonal and oblique. Orthogonal rotations (Varimax and Quartrimax) do not expect any correlation between the variables. Oblique rotations (Oblimin and Promax) produce a pattern matrix that contains the item loadings and a factor correlation matrix that includes the correlation between factors [71]. In this study, Promax rotational method was implemented since this is a kind of psychological index which processes correlations between factors. "Promax is expedient because of its speed in larger databases. It involves raising the loadings to a power of four, ultimately resulting in greater correlations among the factors and achieving a simple structure" [72]. Our study used the Promax rotation with suppressing small coefficients with an absolute value of 0.04. The resulting factor structure was unidirectional, and the homogeneity was prominently illustratable.

Factor loadings and cross-loadings were of interest, and generally, the loadings at the level of 0.3 are considered acceptable according to the literature [52]. Moreover, cross-loading is when an item loads at 0.32 or higher on two or more factors [68]. In the present study, the problem of cross-loading was removed when the rotational power was set at 0.4 under Promax rotation. All the factors were loaded clearly with a factor loading > 0.7, except for one variable, as explained above. Thus, the developed and validated instrument of AODTII has met the standard criteria for its validity and reliability in assessing the ODT impact on adolescents in the Colombo district, Sri Lanka. One limitation of the study samples is their generalisability for the whole country. The study samples for PCA, EFA and CFA were taken from the Colombo district and may not represent the rural areas.

A composite scale was transformed to represent the cumulative impact of all four constructs. Each question in each domain was weighted according to their covariance obtained in the PCA and summed to obtain the composite score [73]. The cut-off levels for the perception of impact were divided into three categories per the distribution of the composite scores in four quartiles. The significant impact was assigned for the values between Q1 and Q3; the Less significant impact up to Q1 value and the Highly significant Impact beyond Q3 value. Therefore, when the AODTII is administered, and the values are obtained according to the formula in Fig. 2, This weighted composite score calculation method is available in the literature [73].

### Psychometric properties of the AODTII

#### Validity of the AODTII

To obtain the construct validity of a scale, it is common practice to assess the factor structure by confirmatory factor analysis (CFA) [74]. The AMOS Software Version 29 Graphics were used to demonstrate the construct validity by comparing the observed covariance matrix to estimate the population covariance matrix. Technically, it was expected to minimise the differences between the estimated and observed matrices. Many indices currently available in the literature have yet to assess the construct validity in the development stage using a separate study sample. Some authors have explained that it was absent due to small numbers in diagnostic categories [57]. Some authors used other developed and validated related instruments to measure the construct validity [41]. However, the dilemma in assessing the construct validity of AODTII was the absence of another 'gold standard' tool to be compared. On the other hand, developing a new tool would be useless if it were present.

Thus, the construct validity of AODTII was assessed using the original factor structure with 12 variables and four factors which did not change after the EFA. The study to assess the CFA was done in a separate study sample without contaminating the EFA sample, as explained in the methodology section.

The minimum sample size needed for CFA is also a dilemma in the literature. Large sample sizes were advocated to be avoided since, by default, a relationship might come to light. According to many scholars, the rule of thumb was 100 subjects or a 'five to fifteen subjects per a variable' ratio [52, 54, 75]. In this research, the sample size was taken as 132. The adequacy of sample size was ensured by the KMO measure of sampling adequacy and Bartlette's test of sphericity [71]. The KMO test's cut-off value was 0.6; in the current study, it was 0.734. Bartlett's test showed a significant value, and sampling adequacy was ensured.

Absolute, relative and Parsimony fit indices assessed the model fit statistics. Generally, a structural equation model is a complex composite statistical hypothesis. It consists of two main parts: The measurement and path models [76]. The construct validity of the AODTII was demonstrated using the numerous model fit indices described in Tables 7 and 8. According to the literature, an RMSEA value less than 0.05 corresponds to a "good" fit, and an RMSEA less than 0.08 corresponds to an "acceptable" fit. The four-factor model of the AODTII obtained the RMSEA value of 0.054. Thus, it had an acceptable level of fit. The goodness of Fit Index (GFI), widely explained as another fit index, obtained the value of 0.931. According to the literature, the desired level was > 0.09 [77]. These authors used this index in 15 of their research-work papers. Regarding Relative and Parsimony fit indices CFI and PGFI values show good model fit with values denoting 0.957 and 0.561, respectively. All these indices ensure that the AODTII has good construct validity. As the AODTII has been validated using robust methodology based on the samples from Colombo district, the internal validity of the index is well established for the setting. However, its external validity for a broader context should be explored further with validation studies. Moreover, further research is recommended to improve its translational value.

### Reliability of the AODTII

The Cronbach's alpha of this 12-item index was 0.78 (CI = 0.630 – 0.921; *p* < 0.01). Moreover, it was assessed separately for all the domains, and the values were more than 0.7, which exceeded Nunnally's criteria of 0.7 [78]. Thus, the AODTII was assured in terms of reliability. The higher value indicated that all the subdomains measure the same construct. The fit of a specific variable to the full scale was assessed by deleting the item and looking for the alpha value of the scale. It was evident that, by omitting any of the 12 items, Cronbach's alpha value did not achieve any improvement. Thus, the internal consistency of the AODTII was further verified.

The corrected item-total correlation coefficient ranged from 0.250 to 0.557. Thus, there was no variable to be removed from the index as all the values exceeded the minimum of 0.2, which was the level at which an item is selected for an instrument. Thus, the homogeneity of the instrument was ensured [78].

## Conclusions

The 12-item AODTII has been established as the first condition-specific dependable, and legitimate instrument for gauging the perceived impact of treated and untreated sports-related oro-dental injuries among adolescents in Sri Lanka. The index comprises 12 items aggregated under four factors of social Impact mingled with peer pressure, physical Impact, the Impact caused by the oral health system and the impact due to acceptance of unmet dental treatment needs. This could have implications for its application in other countries. However, additional research is necessary to enhance the practicality of this innovative index. Additionally, the index has the potential to function as a patient-centred communication tool, a clinical adjunct and an advocacy tool. However, it requires the feedback of end-users to be fully supported.

## Data Availability

The datasets analysed during the current study are available from the corresponding author upon reasonable request.

## Abbreviations

AODTII: Adolescent Oro Dental Trauma Impact Index
AGFI: Adjusted Goodness of Fit Index
AMOS: Analysis of Moment Structures
CFA: Confirmatory Factor Analysis
CFI: Comparative Fit Index
CI: Confidence Interval
CPQ: Child Perception Questionnaires
CVR: Content Validity Ratio
DHQ: Dental Health Questions from Rand Health Insurance Company
DIDL: Dental Impact on Daily Living
DIP: Dental Impact Profile
EFA: Exploratory Factor Analysis
FGD: Focus Group Discussions
GFI: Goodness of Fit Index
GOHAI: Geriatric Oral Health Assessment Index
HRQoL: Health-Related Quality of Life
KII: Key Informant Interviews ODT- Oro Dental Trauma
KMO: Kaiser–Meyer–Olkin
ICC: Interclass Correlation Coefficient
NFI: Non-Normed Fit Index
OHIP: Oral Health Impact Profile
OHRQoL: Oral Health-Related Quality of Life
PCA: Principal Component Analysis
PGFI: Parcimony Goodness of Fit Index
PNFI: Parcimony Normed Fit Index
QoL: Quality of Life
RMSEA: Root Mean Square Estimate of Approximation
SIDD: Social Impact of Dental Diseases
SRMR: Standard Root Mean Square Residual
UDTN: Unmet Dental Treatment Need
