# Correction to: Development and validation of a novel index to assess the perceived impact of sports-related oro-dental trauma among adolescents: findings from Sri Lanka

**DOI:** 10.1186/s12903-023-03170-0

**Published:** 2023-07-06

**Authors:** Iresha Udayamalee, Hemantha Amarasinghe, Ping Zhang, Newell Johnson

**Affiliations:** 1 Health Promotion Bureau, Ministry of Health, Colombo, Sri Lanka; 2 School of Medicine and Dentistry, Griffith University, Gold Coast Campus, Queensland, Australia; 3 Faculty of Dental Sciences, University of Jayawardhanapura, Colombo, Sri Lanka; 4 Menzies Health Institute, Griffith University, Gold Coast Campus, QLD Australia; 5 Faculty of Dentistry, Oral and Craniofacial Sciences, King’s College, London, UK

**Correction: BMC Oral Health (2023) 23:388**

10.1186/s12903-023-03097-6

In this article, the affiliation details of authors Iresha Udayamalee and Newell Johnson were incorrect. It has been corrected with this correction.

The original article has been corrected.

